# Acute fibrinolysis shutdown occurs early in septic shock and is associated with increased morbidity and mortality: results of an observational pilot study

**DOI:** 10.1186/s13613-019-0499-6

**Published:** 2019-01-30

**Authors:** Felix Carl Fabian Schmitt, Vasil Manolov, Jakob Morgenstern, Thomas Fleming, Stefan Heitmeier, Florian Uhle, Mohammed Al-Saeedi, Thilo Hackert, Thomas Bruckner, Herbert Schöchl, Markus Alexander Weigand, Stefan Hofer, Thorsten Brenner

**Affiliations:** 1 Department of Anesthesiology, Heidelberg University Hospital, 110, Im Neuenheimer Feld, 69120 Heidelberg, Germany; 2 Department of Internal Medicine I and Clinical Chemistry, Heidelberg University Hospital, Heidelberg, Germany; 3 German Centre for Diabetes Research (DZD), Neuherberg, Germany; 4 Bayer AG, Cardiovascular Research, Wuppertal, Germany; 5 Department of General, Visceral and Transplantation Surgery, Heidelberg University Hospital, Heidelberg, Germany; 6 Institute of Medical Biometry and Informatics, University of Heidelberg, Heidelberg, Germany; 7 Department of Anesthesiology and Intensive Care Medicine, AUVA Trauma Centre Salzburg, Academic Teaching Hospital of the Paracelsus Medical University, Salzburg, Austria; 8 Clinic for Anesthesiology, Intensive Care and Emergency Medicine I, Westpfalz Hospital, Kaiserslautern, Germany; 9 Institute for Experimental and Clinical Traumatology, AUVA Research Centre, Vienna, Austria

**Keywords:** Fibrinolysis shutdown, Rotational thromboelastometry, Point-of-care testing, Thrombin–antithrombin, Plasminogen activator inhibitor 1, Tissue plasminogen activator, Thrombin generation assay

## Abstract

**Background:**

Septic coagulopathy represents a very dynamic disease entity, tilting from initial hypercoagulability towards a subsequent hypocoagulable disease state, entitled overt disseminated intravascular coagulation. Acute fibrinolysis shutdown has recently been described to be a crucial component of initial hypercoagulability in critically ill patients, although the underlying pathomechanisms, the specific temporal kinetics and its outcome relevance in patients with sepsis remain to be determined.

**Methods:**

In total, 90 patients (30 with septic shock, 30 surgical controls and 30 healthy volunteers) were enrolled. Blood samples were collected at sepsis onset or prior and immediately after the surgical procedure as well as 3 h, 6 h, 12 h, 24 h, 48 h and 7 d later, whereas blood samples from healthy volunteers were collected once. Besides viscoelastic and aggregometric point-of-care testing (POCT), enzyme-linked immunosorbent and thrombin generation assays and liquid chromatography–mass spectrometry-based measurements were performed.

**Results:**

As assessed by viscoelastic POCT, fibrinolysis shutdown occurred early in sepsis. Significant increases in tissue plasminogen activator had no effect on thromboelastometrical lysis indices (LIs). Contrariwise, plasminogen activator inhibitor-1 was already significantly increased at sepsis onset, which was paralleled by significantly increased LIs in patients suffering from septic shock in comparison with both control groups. This effect persisted throughout the 7-day observation period and was most pronounced in severely ill as well as non-surviving septic patients. Thromboelastometrical LI, therefore, proved to be suitable for early diagnosis [e.g. LI 45 min: area under the curve (AUC) up to 0.933] as well as prognosis (e.g. LI 60 min: AUC up to 1.000) of septic shock.

**Conclusions:**

Early inhibition of plasminogen activation leads to acute fibrinolysis shutdown with improved clot stability and is associated with increased morbidity and mortality in septic patients.

*Trial registration* This study was approved by the local ethics committee (Ethics Committee of the Medical Faculty of Heidelberg; Trial-Code No. S247-2014/German Clinical Trials Register (DRKS)-ID: DRKS00008090; retrospectively registered: 07.05.2015). All study patients or their legal representatives signed written informed consent.

**Electronic supplementary material:**

The online version of this article (10.1186/s13613-019-0499-6) contains supplementary material, which is available to authorized users.

## Background

Given the pathophysiological relationship between inflammation and coagulation, blood coagulation disorders are very common in septic patients [1, 2]. However, septic coagulopathy represents a very dynamic disease entity, tilting from initial hypercoagulability towards a hypocoagulable disease state at later stages due to an excessive consumption of coagulation factors. Overt disseminated intravascular coagulation (overt DIC) represents the most serious form of hypocoagulable septic coagulopathy and is associated with an increased morbidity as well as mortality [3]. However, due to the lack of specific tests, most hypercoagulable blood coagulation disorders (which can be summarized as non-overt DIC disease states) remain unidentified and the diagnosis of overt DIC is currently based on questionable scoring systems [such as the International Society on Thrombosis and Haemostasis (ISTH) score and the Japanese Association for Acute Medicine (JAAM) DIC score], which are both associated with relevant weaknesses. For example, the incidence of an overt DIC varies between 29 and 61% due to doubtful diagnosis criteria as well as differing calculation bases within the two scoring systems [4]. Accordingly, new approaches for a more sophisticated and stage-specific assessment of septic coagulopathy, comprising initial hypercoagulability as well as subsequent hypocoagulable disease states, are absolutely warranted. From a pathophysiological point of view, septic coagulopathy is initially hallmarked by an excessive expression of tissue factor (TF), an inhibition of anticoagulant factors and a so-called fibrinolysis shutdown [3, 5]. Particularly, the phenomenon of fibrinolysis shutdown (as assessed by an increased thromboelastometrical LI) has already been identified as a crucial component of coagulopathy in critically ill patients, which was clearly associated with increased mortality for example in trauma patients [6]. However, detailed information about the underlying pathomechanisms, the specific temporal kinetics and its outcome relevance in patients suffering from sepsis or septic shock is still lacking, so that additional well-designed clinical studies have to be demanded.

The aims of the presented study were therefore (1) to evaluate the pro- as well as anticoagulatory responses in septic coagulopathy and (2) to assess the detailed pathophysiology, kinetics and diagnostic and outcome relevance of fibrinolysis shutdown in patients suffering from sepsis by the use of modern viscoelastic and aggregometric point-of-care testing (POCT) devices as well as routine coagulation tests and elaborate laboratory methods.

## Methods

### Study participants, sample collection, DIC score and VTE prophylaxis

This prospective, observational, clinical study was approved by the local ethics committee (Ethics Committee of the Medical Faculty of Heidelberg; Trial-Code No. S247-2014/German Clinical Trials Register (DRKS)-ID: DRKS00008090). All study patients or their legal representatives signed written informed consent. Between November 2014 and May 2016, 90 patients in three groups were enrolled in the study: (1) 30 patients with septic shock (according to the 2001 SCCM/ESICM/ACCP/ATS/SIS International Sepsis Definitions Conference (*Sepsis*-*2*) [7]) following major abdominal surgery, (2) 30 patients undergoing major abdominal surgery with an uncomplicated course and (3) 30 age-matched healthy volunteers. The following criteria were defined as exclusion criteria: chronic inflammatory diseases, primary or acquired platelet dysfunctions, therapy with oral anticoagulants, pre-existing bleeding disorders, pregnancy or an age of younger than 18 years. Patients with septic shock were re-evaluated for survival at 30 days after enrolment in the study. The diagnosis of DIC was based on the validated score for overt DIC developed by the ISTH [8]. Relevant baseline data were collected at study inclusion in all study groups. In patients suffering from septic shock, blood samples were collected at sepsis onset as well as 3 h, 6 h, 12 h, 24 h, 48 h and 7 days afterwards. Blood samples from the surgical group were collected prior to surgery (pre), immediately after the surgical procedure (onset) and 3 h, 6 h, 12 h, 24 h, 48 h and 7 days later. Viscoelastic (ROTEM^®^) and aggregometric (Multiplate^®^) POCT was performed at all timepoints, whereas clinical data, routine blood parameters and plasma samples for thrombin generation and enzyme-linked immunosorbent assays as well as liquid chromatography–mass spectrometry (LC–MS)-based measurements were only collected at pre (in surgical patients), onset (in patients with septic shock and surgical patients) and 24 h, 48 h and 7 d later. In the healthy control group, blood samples and clinical data were collected only once. For prophylaxis of venous thromboembolism (VTE), patients following major abdominal surgery received either intravenously (i.v.) administered low-dose (10.000 IU/day) unfractionated heparin (UFH) or subcutaneously (s.c.) administered low molecular weight heparin (LMWH; 40 mg enoxaparin/day) starting from 6 h after the end of surgery. In patients suffering from septic shock, VTE prophylaxis was performed with i.v. low-dose (10.000 IU/day) UFH. None of the volunteers in the control group received any anticoagulants.

### Viscoelastic POCT

Viscoelastic POCT in citrated whole blood was performed with ROTEM delta^®^ (Tem International GmbH, Germany) restricted to ex-TEM^®^ test, evaluating the extrinsic coagulation cascade. The following test parameters were recorded: (1) the clotting time (CT) represents the time needed from starting the test until the clot begins to rise. Depending on the test used, a prolongation of the CT may result from a consumption of coagulation factors or the administration of anticoagulants. Contrariwise, shortening of CT represents an indicator for hypercoagulability. (2) The clot formation time (CFT) is described to be the time from CT (clot begins to rise) until a clot firmness of 20 mm has been reached. The CFT is mainly dependent on the platelet count as well as the fibrinogen level. A reduction in each factor is able to result in a prolongation of the CFT. (3) The lysis index (LI) is the percentage of the remaining clot firmness after 45 min (LI 45) and 60 min (LI 60), indicating the amount of intrinsic fibrinolytic activity. All ROTEM^®^ measurements were performed in duplicate.

### Aggregometric POCT

Aggregometric POCT in hirudin-anticoagulated whole blood was performed on the multiple electrode aggregometry (MEA) platelet function analyser (Multiplate^®^; Roche Diagnostics GmbH, Germany). Test cells of this device incorporate a duplicate sensor for acceptance sampling. Platelets were stimulated in various ways: (1) via arachidonic acid (ASPI test; Roche Diagnostics GmbH, Germany), (2) the ADP receptor (ADP test, Roche Diagnostics GmbH, Germany), (3) the thrombin receptor under the use of thrombin receptor-activating peptide-6 (TRAP test; Roche Diagnostics GmbH, Germany) and (4) via the collagen receptor (COL test, Roche Diagnostics GmbH, Germany). Once initiated, platelet aggregation was allowed to run up to 6 min, and the area under the curve (AUC) was calculated.

### ELISA measurements

Plasma levels of thrombin–antithrombin complex (TAT), plasminogen activator inhibitor 1 (PAI-1), free tissue plasminogen activator (tPA) and total tissue plasminogen activator (total tPA) were measured using enzyme-linked immunosorbent assay (ELISA) kits according to the manufacturer’s instructions (Assaypro, St. Charles, USA). ELISA measurements were performed in duplicate.

### TGA measurements

The thrombin generation assay (TGA; calibrated automated thrombogram; all reagents from Diagnostica Stago) was conducted with a validated method [9], in which thrombin generation was triggered in 80 µL of citrated platelet-poor plasma (PPP) by addition of a PPP reagent solution containing TF (final concentration 5 µM) and phospholipids (final concentration 4 µM). After 5-min incubation time at 37 °C, 20 µL of the Flu-Ca reagent was added. The activity of the thrombin generated resulted in conversion of a fluorogenic substrate. Fluorescence was measured continuously for 90 min with a MTP fluorometer (Diagnostica Stago), which relates the fluorescence to a thrombin calibrator. TGA parameters (lag time, time to peak and peak height) were derived with Thrombinoscope software (Thrombinoscope, Maastricht, The Netherlands).

### LC–MS measurements

Extraction of 6-keto-prostaglandin F1α (PG F1α) and 11-dehydro-thromboxane B_2_ (11d-TX B_2_) by liquid–liquid extraction was performed as described previously [10]. The quantification via LC–MS/MS was performed with minor modifications [10]. Calibration range for each compound was as follows: PG F1α at 0.05, 0.1, 0.2, 0.4 and 0.8 pmol for each calibrator per injection; 11d-TX B_2_ at 0.05, 0.1, 0.2, 0.4 and 0.8 pmol for each calibrator per injection.

For the detection of PG F1α and 11d-TX B_2_, the parameters for electrospray ionization were set as follows: capillary voltage—2.0 kV; desolvation temperature—300 °C; desolvation gas flow—850 L/h; source temperature—150 °C; cone gas flow—250 L/h; collision gas flow—0.15 mL/min; and nebulizer gas flow—5 bar. Cone and collision voltage was optimized for each compound separately: for PG F1α: retention time (*R*_*t*_) 2.90 (min), mass transition (MRM) 369.1 > 163.2 (m/z), cone voltage (CV) 35 (V) and collision voltage (CE) 26 (V); and for 11d-TX B_2_: *R*_*t*_ 3.21 (min), MRM 367.1 > 305.3 (m/z), CV 35 (V) and CE 15 (V).

### Statistics and electronic database

Study data were entered into an electronic database (Microsoft^®^ Excel 2011, Microsoft Corporation, Redmond, USA) and evaluated using SPSS software (Statistical Product and Services Solutions, version 24.0, SPSS Inc., Chicago, USA). Categorical data were summarized by means of absolute and relative frequencies. Quantitative data were summarized using medians (with quartiles). Wherever appropriate, data were visualized using line charts. The Kolmogorov–Smirnov test was applied to check for normal distribution. Due to non-normally distributed data, nonparametric methods for evaluation were used (categorical data: Chi-square test/continuous data: Kruskal–Wallis test as a global testing procedure and Mann–Whitney test for pairwise comparisons as well as Friedman test and Wilcoxon test for in-group comparisons). Furthermore, a receiver operating characteristic (ROC) analysis was performed with suitable parameters, in order to create cut-off values to determine the diagnostic or prognostic value of each parameter with regard to the diagnosis of sepsis and/or the estimation of outcome. A *P* value < 0.05 was considered statistically significant. Due to the explorative nature of the present investigation, no alpha adjustment was performed.

## Results

### Patient characteristics

All patients of the surgical control group underwent major abdominal surgery with an uncomplicated course. Patients in the septic group underwent major abdominal surgery as well, but suffered from septic shock due to a medical or surgical complication and were therefore hallmarked by a high disease severity at study inclusion as assessed by the APACHE II as well as SOFA scores. Relevant data of all study groups are presented in detail in Table 1.

**Table 1:** Patients’ characteristics

*Healthy group*	
Age (years)	60 (56.3–65.0)
Gender (male)	20 (66.7%)
ASA status I; II; III; IV; V	3 (10.0%); 16 (53.3%); 11 (36.7%); 0; 0
*Surgical group*	
Age (years)	60 (55.5-73.5)
Gender (male)	21 (67.7%)
ASA status I; II; III; IV; V	2 (6.7%); 16 (53.3%); 12 (40.0%); 0; 0
Site of surgery (double naming feasible)	
Liver	8 (25.8%)
Pancreas	14 (45.2%)
Gastrointestinal	23 (74.2%)
*Septic group*	
Age (years)	63.5 (53.8–73.0)
Gender (male)	23 (76.7%)
SOFA score (at Onset)	13.0 (12.0–14.5)
APACHE II (at Onset)	28.0 (25.3–32.8)
Primary site of infection/septic focus (double naming feasible)	
Surgical focus	28 (93.3%)
Medical focus	
Pneumonia	5 (16.7%)
Urinary tract infection	1 (3.3%)
Outcome	
Survivor 30 days	18 (60.0%)

Data are presented by median and interquartile range (Q1–Q3) or number and percentage

*ASA status* American Society of Anesthesiologists physical status classification system, *SOFA score* sepsis-related organ failure assessment  score, *APACHE II score* Acute Physiology and Chronic Health Evaluation II score

### Infection

Inflammatory and infection marker levels (such as leucocyte count, C-reactive protein (CRP) and procalcitonin (PCT)) were shown to be increased in surgical and septic patients (Table 2).

**Table 2:** Laboratory parameters, ELISA and LC–MS measurements

Parameter	Baseline	Pre	Onset		24 h		48 h		7d	
	Healthy	Surgical	Surgical	Sepsis	Surgical	Sepsis	Surgical	Sepsis	Surgical	Sepsis
Leucocytes (1/nL)	6.16 (5.11–7.58)	6.14 (4.53–8.42)	12.18 (9.71–14.89)	19.21 (7.74–27.31)	11.71 (8.96–13.95)	19.86 (12.51–33.36)	9.51 (7.64–12.00)	19.95 (12.31–30.84)	10.35 (7.65–13.30)	14.21 (9.52–20.05)
			0.039*		< 0.001***		< 0.001***		0.115	
CRP (mg/L)	2.00 (2.00–2.85)	2.00 (2.00–6.65)	2.70 (2.00–9.85)	218.90 (144.05–269.08)	133.70 (90.63–157.40)	263.25 (159.75–320.30)	143.30 (112.10–162.30)	179.00 (116.25–293.05)	68.30 (29.45–130.70)	120.90 (72.30–158.10)
			< 0.001***		< 0.001***		0.041*		0.150	
PCT (ng/mL)	0.05 (0.05–0.05)	0.05 (0.05–0.07)	0.09 (0.05–0.19)	17.54 (1.68–48.18)	1.08 (0.43–2.18)	11.96 (3.27–35.07)	0.78 (0.36–1.49)	8.99 (1.77–28.69)	0.11 (0.10–0.81)	1.03 (0.47–1.45)
			< 0.001***		< 0.001***		< 0.001***		0.008**	
TAT (ng/mL)	3.30 (2.40–6.60)	9.90 (6.33–11.35)	9.90 (6.68–11.70)	12.85 (11.16–14.83)	9.23 (5.91–13.38)	12.53 (10.84–15.28)	10.30 (5.35–12.95)	13.73 (11.28–17.48)	10.90 (6.83–13.40)	14.70 (11.63–18.45)
			< 0.001***		0.003**		0.001**		0.021*	
D-dimer (mg/L)	0.37 (0.26–0.57)	0.48 (0.34–0.85)	3.55 (1.85–4.13)	7.59 (4.54–13.85)	3.50 (2.23–4.10)	6.94 (4.16–12.27)	2.41 (1.85–3.38)	6.77 (4.14–11.87)	4.68 (3.78–5.84)	5.47 (4.32–7.43)
			< 0.001***		0.001**		< 0.001***		0.412	
PT index (%)	109.5 (104.70–117.30)	94.00 (88.30–100)	91.00 (78.20–98.75)	68.75 (46.85–76.35)	70.05 (61.30–84.40)	65.80 (53.63–85.65)	94.00 (85.70–100.90)	81.00 (65.75–92.13)	96.80 (89.85–113.80)	81.90 (71.40–91.60)
			< 0.001***		0.304		0.008**		0.004**	
aPTT (s)	24.00 (22.65–25.90)	23.80 (22.65–25.90)	22.00 (20.60–23.95)	32.90 (28.45–37.80)	26.35 (24.53–28.48)	37.80 (31.33–44.53)	26.30 (23.80–28.00)	33.90 (27.65–40.55)	24.50 (21.50–27.05)	27.90 (26.10–35.00)
			< 0.001***		< 0.001***		< 0.001***		0.009**	
Platelet count (1/nL)	249.00 (209.50–275.75)	183.00 (167.50–232.50)	200.00 (172.00–248.50)	181.50 (101.50–229.50)	156.00 (133.00–191.80)	152.5 (59.30–253.00)	156.00 (137.00–197.00)	142.00 (68.00–255.00)	264.00 (246.00–315.50)	258.00 (204.50–377.50)
			0.157		0.408		0.252		0.755	
PG F1α (pg/mL)	22.9 (20.7–48.0)	82.0 (45.8–121.4)	54.9 (28.3–71.2)	278.3 (50.3–591.3)	10.1 (5.2–45.5)	431.0 (117.9–972.2)	7.7 (2.0–27.9)	283.9 (97.7–1165.8)	7.5 (1.2–59.1)	345.2 (48.4–689.0)
			< 0.001***		< 0.001***		< 0.001***		0.016*	
11d-TX B2 (pg/mL)	45.6 (36.9–73.4)	8.3 (5.1–19.8)	9.5 (5.6–14.6)	18.7 (9.6–39.7)	12.6 (7.7–34.2)	48.7 (21.9–159.4)	20.6 (11.8–48.2)	54.4 (28.6–144.0)	13.5 (8.6–41.0)	18.3 (7.9–45.1)
			0.013*		< 0.001***		0.005**		0.893	

Data are presented by median and interquartile range (Q1–Q3)

*TAT* thrombin–antithrombin complex, *INR* international normalized ratio, *aPTT* activated partial thromboplastin time, *CRP* C-reactive protein, *PCT* procalcitonin, *PT index* prothrombin index, *PG F1α* prostaglandin F1α, *11d-TX B2*  11-dehydro Thromboxane B2

A *P* value < 0.05 was considered statistically significant. Concerning symbolism and higher orders of significance: *P < 0.05, **P < 0.01, ***P < 0.001

### Increased intravascular formation of fibrin

**Fig. 1:**
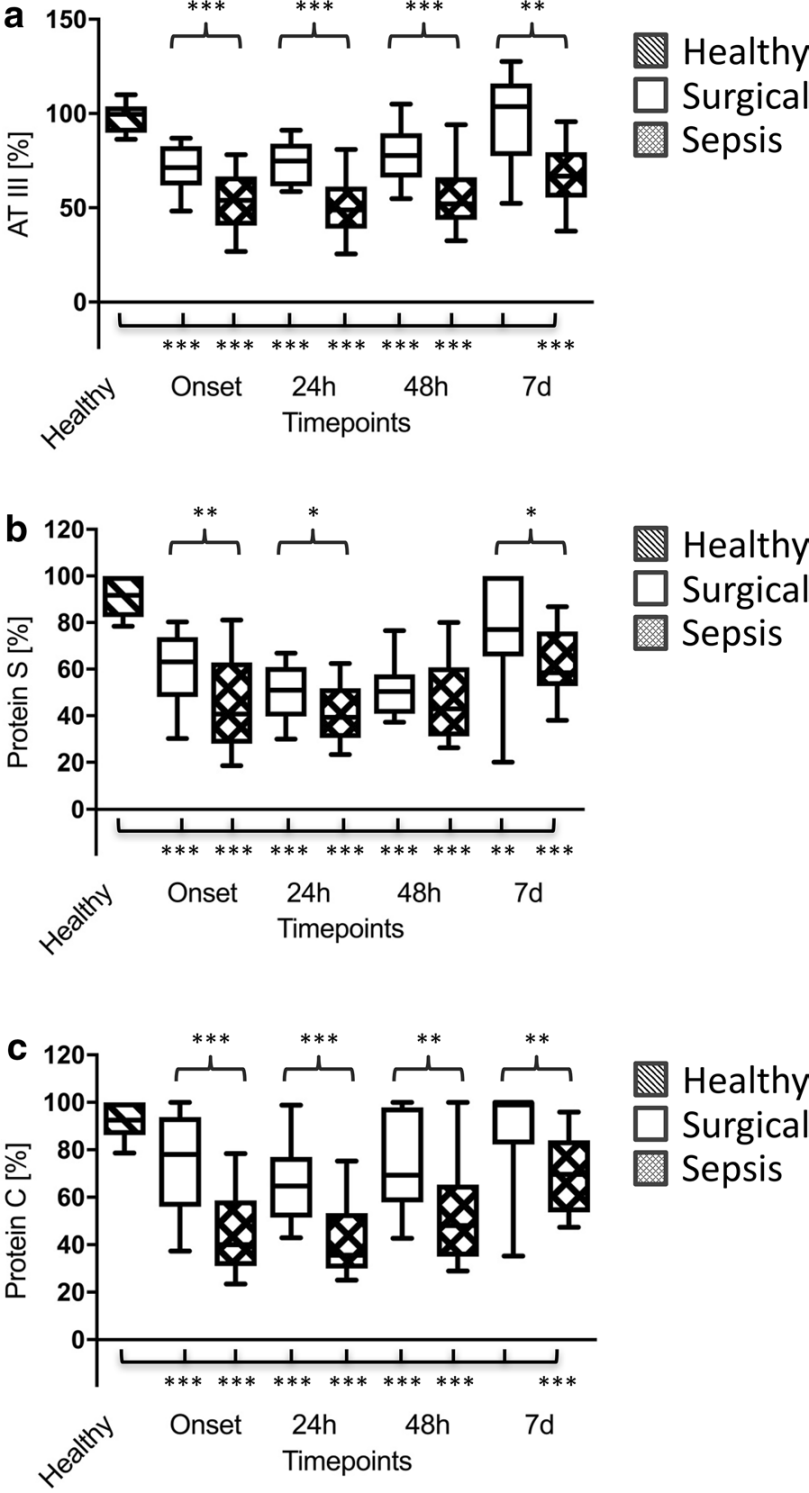
Plasma levels of antithrombin (AT) III (**a**), protein S (**b**) and protein C (**c**) in healthy volunteers (*n* = 30; striped bars), surgical patients (*n* = 30; white bars) and patients with septic shock (*n* = 30; squared bars). Data of patients with septic shock and surgical patients are presented for the timepoints onset (sepsis onset or immediately after the surgical procedure) as well as 24 h, 48 h and 7 d later, whereas blood samples from healthy volunteers were collected only once. Data in box plots are given as median, 25th percentile, 75th percentile with the 10th and 90th percentile at the end of the whiskers. A *P* value < 0.05 was considered statistically significant

The coagulation system was remarkably activated, in both the surgical and septic groups, whereas this effect was most pronounced in septic patients. Accordingly, patients with septic shock showed significantly increased levels of TAT complexes as well as D-dimers in comparison with patients of the surgical group throughout the whole observation period (Table 2). Moreover, anticoagulatory mechanisms, such as antithrombin (AT) III, protein S and protein C, were shown to be significantly impaired in the surgical as well as the septic groups in comparison with healthy volunteers, whereas this effect was again most pronounced in septic patients (Fig. 1a–c).

### Decreased intravascular fibrin degradation

**Fig. 2:**
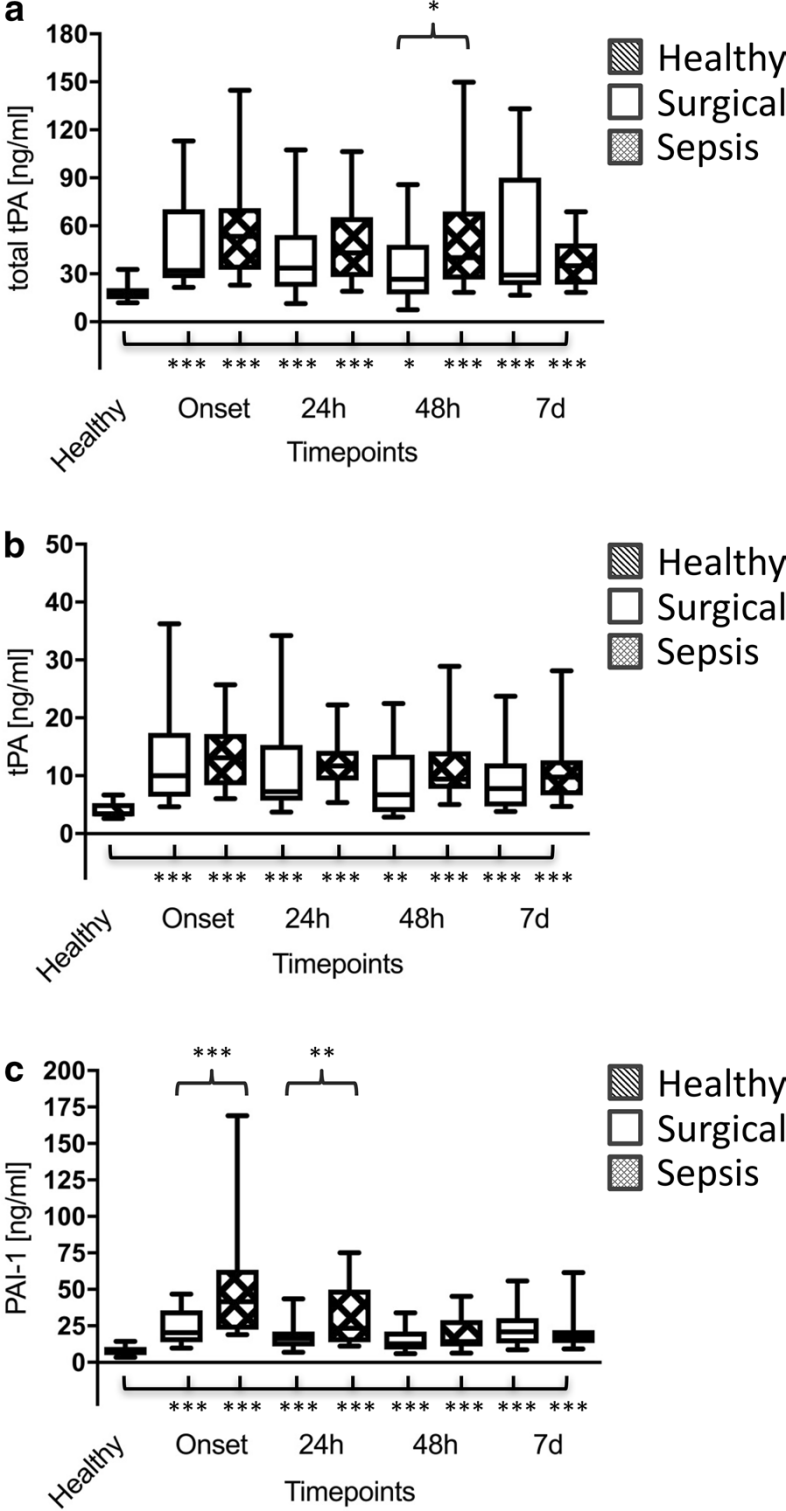
Total thrombin plasminogen activator (total tPA) (**a**), free thrombin plasminogen activator (tPA) (**b**) and plasminogen activator inhibitor-1 (PAI-1) plasma levels (**c**) of healthy volunteers (*n* = 30; striped bars), surgical patients (*n* = 30; white bars) and patients with septic shock (*n* = 30; squared bars). Data of patients with septic shock and surgical patients are presented for the timepoints onset (sepsis onset or immediately after the surgical procedure) as well as 24 h, 48 h and 7 d later, whereas blood samples from healthy volunteers were collected only once. Data in box plots are given as median, 25th percentile, 75th percentile with the 10th and 90th percentile at the end of the whiskers. A *P* value < 0.05 was considered statistically significant

**Fig. 3:**
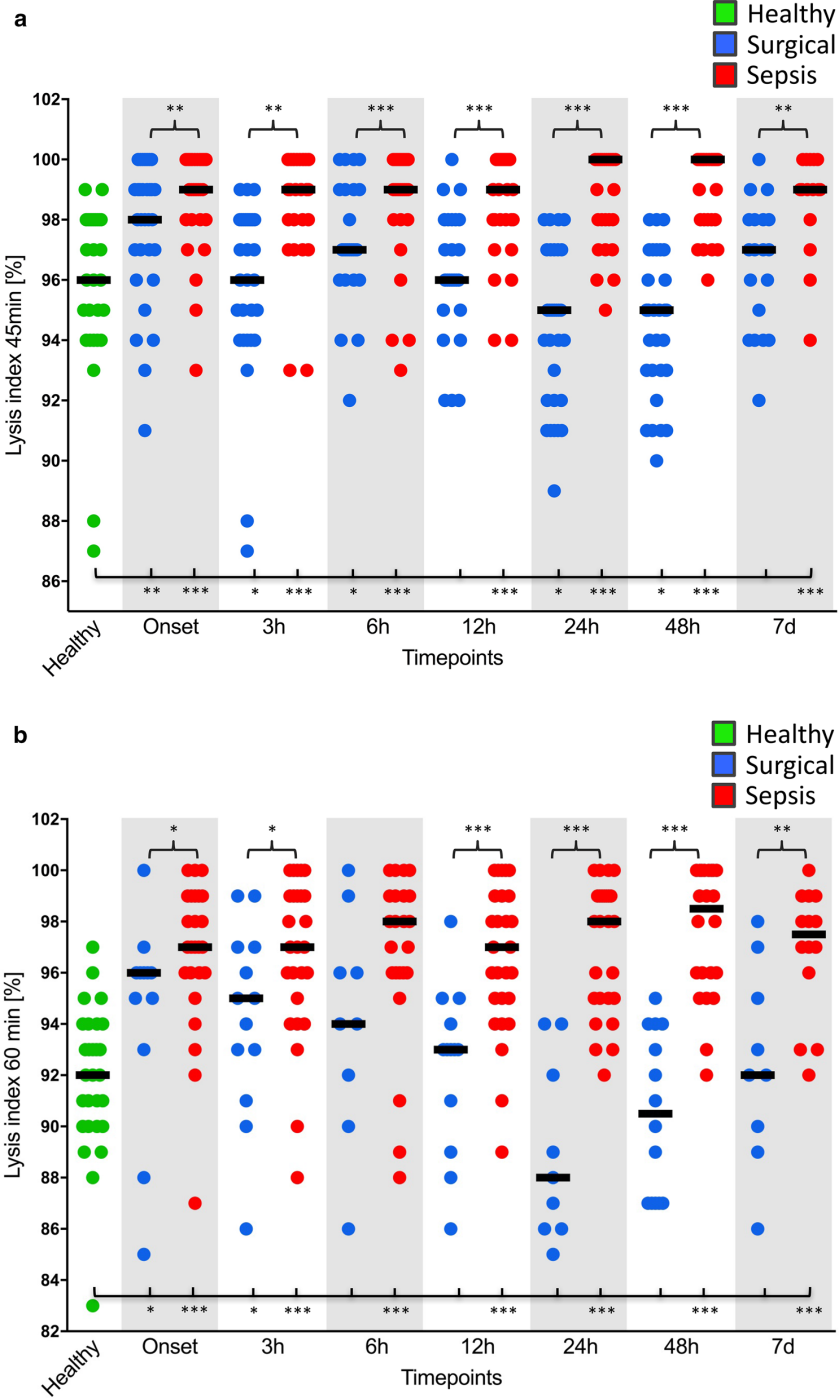
EXTEM lysis indices (LIs) at 45 min (**a**) and 60 min (**b**) in blood samples of healthy volunteers (*n* = 30; green dots), surgical patients (*n* = 30; blue dots) and patients with septic shock (*n* = 30; red dots). Data of patients with septic shock and surgical patients are presented for the timepoints onset (sepsis onset or immediately after the surgical procedure) as well as 3 h, 6 h, 12 h, 24 h, 48 h and 7 d later, whereas blood samples from healthy volunteers were collected only once. A *P* value < 0.05 was considered statistically significant

Plasma levels of total tPA and tPA were significantly increased in both the surgical and septic groups in comparison with healthy volunteers (Fig. 2a, b), which, however, did not result in a reduction in thromboelastometrical LIs (LI 30 min, LI 45 min and LI 60 min) (Fig. 3a, b). On the contrary, plasma levels of PAI-1 were shown to be increased in both the surgical and septic groups in comparison with healthy controls (Fig. 2c), which was paralleled by a significant increase in viscoelastic LIs at 45 min and 60 min. However, this effect was most pronounced in patients with septic shock. Interestingly, LIs at 45 min and 60 min in surgical controls returned to the baseline level within 24 h, whereas fibrinolysis shutdown persisted throughout the whole observation period in patients with septic shock (Fig. 3a, b).

### Consumption of coagulation factors

**Fig. 4:**
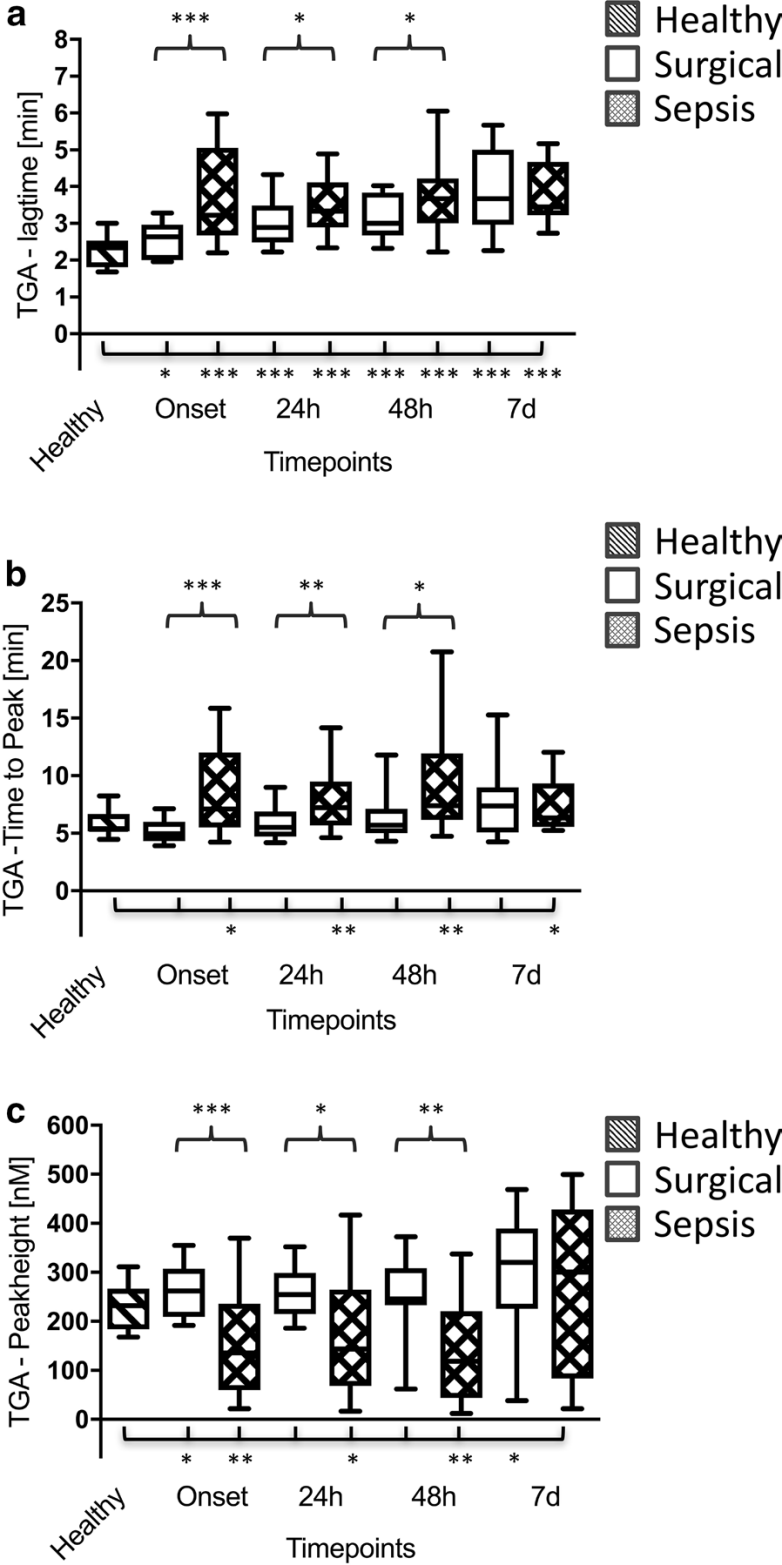
Thrombin generation assay (TGA) lag time (**a**), time to peak (**b**) and  peak height (**c**) in healthy volunteers (*n* = 30; striped bars), surgical patients (*n* = 30; white bars) and patients with septic shock (*n* = 30; squared bars). Data of patients with septic shock and surgical patients are presented for the timepoints onset (sepsis onset or immediately after the surgical procedure) as well as 24 h, 48 h and 7 d later, whereas blood samples from healthy volunteers were collected only once. Data in box plots are given as median, 25th percentile, 75th percentile with the 10th and 90th percentile at the end of the whiskers. A *P* value < 0.05 was considered statistically significant

A consumption of procoagulant factors could be observed in patients suffering from septic shock, as indicated by a decreased PT index and a prolonged activated partial thromboplastin time (aPTT) (Table 2). In line with that, CT values in ex-TEM^®^ were significantly prolonged in the septic group up to 48 h after sepsis onset in comparison with the surgical group (Table 3). Moreover, thrombin generation as assessed by the lag time (Fig. 4a) and the time to peak (Fig. 4b) was significantly prolonged, and peak height (Fig. 4c) was significantly reduced in the septic group as compared to both control groups.

**Table 3:** Viscoelastic and aggregometric POCT

Timepoints	Baseline	Pre	Onset		3 h		6 h		12 h		24 h		48 h		7d	
Groups	Healthy	Surgical	Surgical	Sepsis	Surgical	Sepsis	Surgical	Sepsis	Surgical	Sepsis	Surgical	Sepsis	Surgical	Sepsis	Surgical	Sepsis
*Viscoelastic POCT (EXTEM test)*																
CT (s) (normal range 38–79 s)	57.0 (54.0–60.0)	60.0 (55.5–67.0)	60.0 (55.0–70.0)	81.5 (67.8–91.8)	60.0 (54.5–65.5)	78.0 (70.8–89.8)	59.0 (53.0–66.0)	82.0 (67.0–88.0)	66.5 (61.3–71.0)	82.0 (74.0–90.0)	59.0 (55.0–64.0)	81.0 (69.0–86.0)	62.0 (57.0–69.0)	72.0 (66.8–78.0)	71.0 (64.0–78.0)	76.0 (66.0–82.5)
			< 0.001***		< 0.001***		< 0.001***		< 0.001***		< 0.001***		0.001**		0.358	
LI 45 min (%) (normal range > 85%)	96.0 (94.0–98.0)	97.0 (96.0–98.5)	98.0 (96.0–98.5)	99.0 (98.0–100.0)	97.0 (96.0–99.0)	99.0 (98.0–100.0)	97.0 (96.0–99.0)	99.0 (98.0–100.0)	96.0 (95.0–98.0)	99.0 (98.0–100.0)	95.0 (92.0–97.0)	100.0 (98.0–100.0)	95.0 (93.0–97.0)	100.0 (98.0–100.0)	97.0 (94.5–98.0)	99.0 (98.5–100.0)
			0.032*		0.004**		0.008**		< 0.001***		< 0.001***		< 0.001***		0.002**	
LI 60 min (%) (normal range > 85%)	92.0 (90.0–93.75)	93.0 (92.0–95.0)	96.0 (94.5–96.0)	97.0 (96.0–99.0)	95.0 (93.0–97.0)	97.0 (95.3–99.0)	94.0 (92.0–96.0)	98.0 (96.0–99.0)	93.0 (91.0–94.0)	97.0 (95.0–99.0)	88.0 (86.0–92.0)	98.0 (95.0–99.0)	90.5 (87.0–93.75)	98.5 (96.0– 100.0)	92.0 (90.0–95.0)	97.5 (96.3–98.8)
			0.027*		0.032*		0.079		< 0.001***		< 0.001***		< 0.001***		0.005**	
*Aggregometric POCT*																
ADP test AUC (U*min) (normal range 53–122)	65.0 (52.8–82.8)	63.5 (42.3–83.8)	83.5 (47.0–100.5)	29.0 (19.8–58.8)	58.0 (25.5–92.0)	36.0 (19.0–55.5)	52.0 (37.0–108.0)	34.5 (15.8–60.8)	73.0 (37.0–197.5)	48.0 (18.0–70.0)	58.5 (39.8–80.5)	40.0 (17.0–61.0)	53.0 (34.5–69.5)	27.0 (13.3–44.8)	104.0 (80.0–139.5)	71.0 (31.0–108.0)
			< 0.001***		0.143		0.009**		0.101		0.022*		0.008**		0.059	
ASPI test AUC (U*min) (normal range 75–136)	107.0 (94.8–117.8)	93.5 (77.3–112.8)	78.5 (45.5–104.3)	64.5 (24.3–92.0)	41.0 (28.5–88.5)	57.0 (22.0–105.0)	45.0 (27.0–89.0)	53.0 (27.8–100.8)	55.0 (27.0–75.5)	52.0 (24.0–107.0)	48.5 (19.8–74.3)	58.0(26.0–104.0)	37.5(23.5–58.0)	49.0 (23.8–111.8)	118.0 (66.5–146.5)	102.0 (75.5–143.5)
			0.451		0.564		0.583		0.444		0.91		0.191		0.729	
COL test AUC (U*min) (normal range 46–117)	87.0 (72.0–106.8)	63.5 (49.3–80.3)	63.0 (42.0–98.0)	43.0 (33.3–66.5)	53.0 (32.0–100.0)	58.0 (26.5–116.0)	86.0 (49.0–116.0)	55.5 (22.3–87.0)	94.0 (37.5–119.0)	77.0 (29.0–95.0)	58.5 (29.8–92.0)	56.0 (35.0–88.0)	52.0 (26.5–69.5)	54.0 (29.3–96.0)	91.0 (64.0–110.0)	59.0 (42.5–120.5)
			0.680		0.932		0.062		0.277		1.00		0.406		0.405	
ADP test AUC (U*min) (normal range 53–122)	65.0 (52.8–82.8)	63.5 (42.3–83.8)	83.5 (47.0–100.5)	29.0 (19.8–58.8)	58.0 (25.5–92.0)	36.0 (19.0–55.5)	52.0 (37.0–108.0)	34.5 (15.8–60.8)	73.0 (37.0–197.5)	48.0 (18.0–70.0)	58.5 (39.8–80.5)	40.0 (17.0–61.0)	53.0 (34.5–69.5)	27.0 (13.3–44.8)	104.0 (80.0–139.5)	71.0 (31.0–108.0)
			< 0.001***		0.143		0.009**		0.101		0.022*		0.008**		0.059	

Data are presented by median and interquartile range (Q1–Q3)

*CT* clotting time, *LI* lysis index, *AUC* area under the curve

A *P* value < 0.05 was considered statistically significant. Concerning symbolism and higher orders of significance: *P < 0.05, **P < 0.01, ***P < 0.001

### Impaired platelet function

Although the platelet count did not differ significantly between septic patients and surgical controls, platelet function was shown to be impaired in septic patients as assessed by aggregometric POCT (Table 3). As assessed by LC–MS measurements, PG F1α was significantly increased in septic patients in comparison with both control groups throughout the whole observation period. In parallel, plasma levels of 11d-TX B_2_ were also increased in the septic group up to 48 h after sepsis onset, as compared to the surgical control group (Table 2).

### Subgroup analyses

**Fig. 5:**
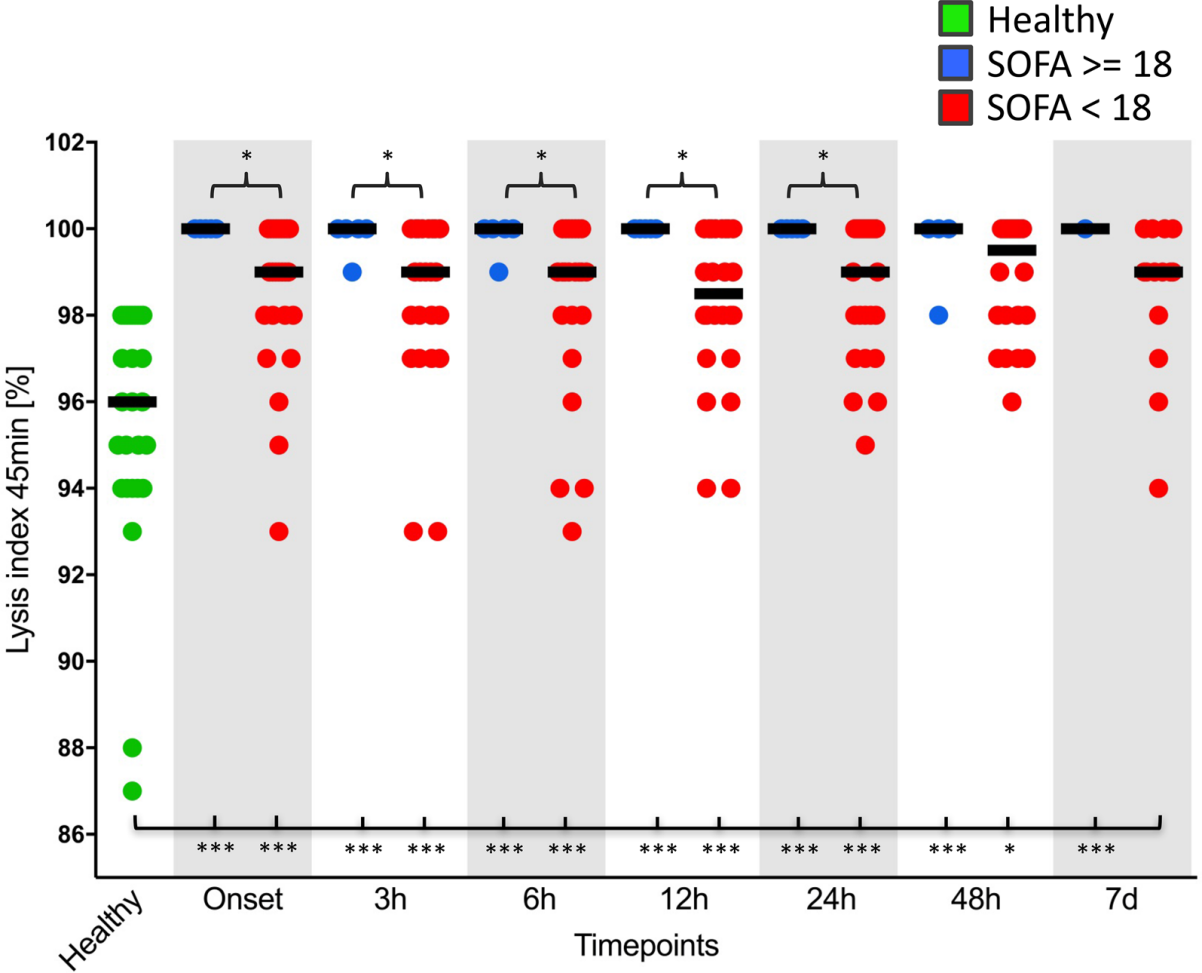
EXTEM lysis index (LI) at 45 min in blood samples of healthy volunteers (*n* = 30; green dots), patients with septic shock and a sepsis-related organ failure assessment (SOFA) score ≥ 18 (*n* = 5 at onset; blue dots) and patients with septic shock and a SOFA score < 18 (*n* = 25 at sepsis onset; red dots). Blood samples from healthy volunteers were collected once, whereas samples from patients with septic shock were collected at sepsis onset as well as 3 h, 6 h, 12 h, 24 h, 48 h and 7 d later. A *P* value < 0.05 was considered statistically significant

An overt DIC could be observed in 43.3% (*n* = 13) of the septic patients and 40.0% (*n* = 12) died within the 30-day observation period. Patients with an overt DIC (*n* = 13) revealed a 23.8% decreased survival as compared to non-overt DIC patients (*n* = 17). Moreover, overt DIC patients were hallmarked by a more pronounced lysis shutdown as well as an accompanying consumption of coagulation factors in comparison with septic patients without DIC and healthy volunteers (e.g. at sepsis onset) (Additional file 1). Further subgroup analyses were performed with septic patients suffering from a high disease severity (SOFA score ≥ 18) as well as non-surviving septic patients (30-day mortality). It could be clearly demonstrated that fibrinolysis shutdown was most pronounced in patients with a SOFA score ≥ 18 in comparison with those septic patients with a SOFA score < 18 (Fig. 5). The same holds true for non-surviving septic patients, in which fibrinolysis shutdown was significantly more pronounced in comparison with surviving septic patients (e.g. increased LI 60 min). As assessed by ROC analysis, the extent of fibrinolysis shutdown (e.g. LI 60 min) proved to be suitable for outcome estimation in patients suffering from septic shock (e.g. at onset: ROC area under the curve (AUC): 0.875 [95% CI 0.602–1.00]; at 3 h: ROC AUC: 1.000 [95% CI 1.000–1.000]). Moreover, platelet function was also shown to be of prognostic value in sepsis, since COX-dependent (ASPI test and COL test), ADP-dependent (ADP test) and TRAP-dependent (TRAP test) stimulation of platelets was significantly diminished in non-surviving septic patients in comparison with surviving septic patients at several timepoints throughout the whole observation period (Additional file 2).

### Heparin effect

Since patients in the septic as well as surgical groups received either UFH (septic group) or enoxaparin (surgical group) for VTE prophylaxis, specifically calibrated anti-Xa activities and thrombin time (TT), which is known to be more sensitive for UFH as compared to the aPTT, were measured in all groups in order to exclude relevant effects of both, UFH or enoxaparin, respectively. However, anti-Xa activities and TT did not differ significantly between the three study groups, strengthening the hypothesis that neither the use of UFH, nor the administration of enoxaparin, had a relevant effect on patients’ coagulation status within the presented investigation (data not shown).

## Discussion

**Fig. 6:**
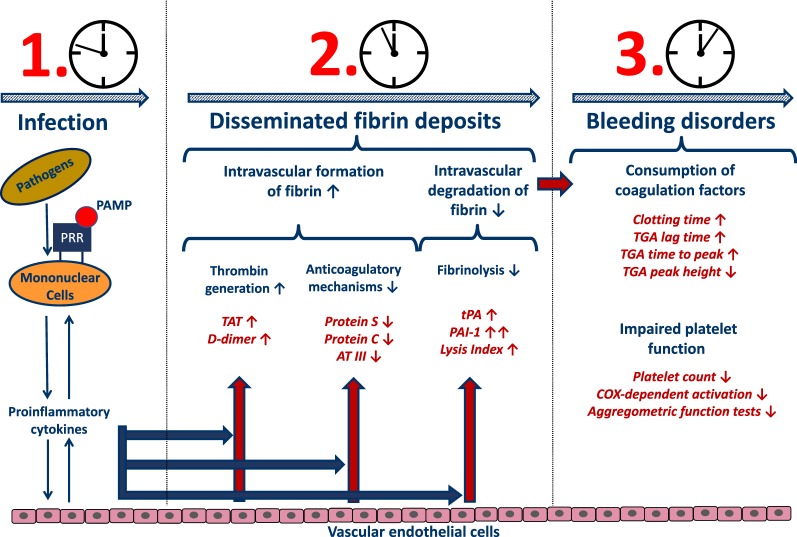
This graphical summary clarifies the most important findings of the presented investigation within the context of septic coagulopathy. *PRR* pattern recognition receptor, *PAMP* pathogen-associated molecular pattern, *AT III* antithrombin III, *TF* tissue factor, *TAT* thrombin–antithrombin complex, *tPA* tissue plasminogen activator, *PAI-1* plasminogen activator inhibitor 1, *TGA* thrombin generation assay, *COX* cyclooxygenase

An intensive crosstalk between the inflammation and coagulation systems is a well-known phenomenon, especially in critically ill patients [11–14]. In septic patients, pathogens trigger the release of a large variety of pathogen-associated molecular patterns [PAMPs; e.g. lipopolysaccharide (LPS)] [15], which are recognized by so-called pattern recognition receptors (PRRs) on the cell surface of several immune cells (e.g. monocytes). Subsequently, a variety of inflammatory mediators as well as large amounts of TF are released. The host response to a pathogen is invariably associated with coagulation activation, representing a prerequisite for sufficient pathogen clearance [11, 14, 16, 17] (graphical summary—Fig. 6).

In line with this hypothesis, TF-mediated generation of thrombin as well as resulting fibrin formation was most pronounced in patients suffering from septic shock within the presented investigation. Accordingly, plasma levels of TAT and D-dimers were significantly increased in septic patients, indicating a compensatory need for ATIII-associated inactivation of thrombin as well as an increased level of fibrin degradation resulting in significantly reduced plasma levels of ATIII in patients with septic shock in comparison with both control groups. In parallel, plasma levels of protein S and protein C were also shown to be significantly reduced. The lack of these anticoagulatory mechanisms represents a well-known phenomenon, especially in critically ill patients suffering from sepsis/septic shock [5], so that anticoagulant therapy was suspected to be of value in these patients [18, 19]. However, the efficacy of this strategy in sepsis remains a matter of dispute, since anticoagulant therapy failed to show a clinical benefit in various clinical trials [20–23]. Of note, more differentiated analyses [24–30] revealed a beneficial effect restricted to septic patients presenting with sepsis-induced DIC and/or suffering from a severe disease state. However, decision-making based on surrogates, such as the DIC or SOFA score, or the use of an unreflected time frame as the only decision criterion may also result in incorrect or inadequate treatment. Although viscoelastic POCT was most frequently used for early detection of severe coagulation disorders in cases of severe or life-threatening bleeding events [31], POCT was also shown to be suitable for the detection of hypercoagulability (e.g. due to hypofibrinolysis or accelerated thrombin formation in various disease states including sepsis) [5, 32, 33]. Within this context, viscoelastic POCT may help to identify hypercoagulable patients likely to benefit from anticoagulant treatment.

Due to its pivotal role within the coagulation cascade, a more detailed analysis of thrombin homoeostasis in septic coagulopathy would be of great value [16]. Using an experimental pig model of LPS-induced sepsis, Schöchl et al. were able to show that endotoxaemia resulted in a significant acceleration of the non-activated TEM (na-TEM^®^) CT very early after the end of LPS infusion (at 2 h following LPS administration) [33, 34], which was not reflected by routine coagulation tests. However, one has to keep in mind that this effect might only be of transient nature and can completely turn into the opposite at later stages. As described by Schöchl et al., the initial decrease in CT in na-TEM^®^ was followed by a significant increase in CT in na-TEM^®^, representing a sign for hypocoagulability at later stages. In line with these findings, CTs in both ex-TEM^®^ and in-TEM^®^ in patients of the septic group were shown to be significantly prolonged already at the clinical onset of sepsis, indicating a hypocoagulable disease state. In parallel, TGA measurements also revealed clear signs for a consumption of coagulation factors in septic patients, as assessed by a prolonged lag time, a prolonged time to peak and a decreased peak height. Therefore, clot formation seems to be impaired at the timepoint of study inclusion, representing the clinical onset of sepsis.

Besides conversion of fibrinogen to fibrin, thrombin also closely interacts with the complement system, since it is able to cleave C3 to C3a and C3b and C5 to C5a and C5b, thus amplifying the activation of the complement system. In addition to its well-established role in inflammation, C5a enhances blood thrombogenicity not only through the upregulation of TF (e.g. on endothelial cells, neutrophils and monocytes) but also via PAI-1 expression on various cell types (e.g. basophils and mast cells) [35]. TF expressed on various cells or released from injured cells initiates the physiologically most important TF (extrinsic) pathway, whereas PAI-1 is known to be a strong inhibitor of the fibrinolytic system. PAI-1-mediated fibrinolysis shutdown has already been described in a pig model of endotoxin-induced DIC [33] as well as in human sepsis [36]. We were able to support these findings within the presented investigation. PAI-1 levels were already increased significantly at sepsis onset, which was paralleled by significantly increased LIs in patients suffering from septic shock in comparison with both control groups. Therefore, thromboelastometrical LIs (e.g. LI 45 min) proved to be reliable biomarkers for early identification of patients suffering from sepsis or septic shock as already previously described [5, 37]. The observed fibrinolysis shutdown persisted throughout the 7-day observation period and was most pronounced in non-surviving septic patients as well as in those septic patients suffering from multiorgan failure, as assessed by high disease severity scores (SOFA). Accordingly, thromboelastometrical LIs (e.g. LI 60 min) were additionally shown to be reliable prognostic biomarkers for early identification of high-risk sepsis patients as already described by several other investigations [5, 38, 39]. The same holds true for trauma patients, in which fibrinolysis shutdown can be observed frequently and is also associated with a worse outcome [6]. Most interestingly and in line with results of Schöchl et al. [33], the observed increases in PAI-1 were paralleled by significant increasing plasma levels of total tPA and tPA in septic patients, which, however, had no effect on thromboelastometrical LIs (LI 30 min, LI 45 min and LI 60 min).

Platelets represent an essential element of primary haemostasis [40]. Besides, thrombocytopenia has been described to be a reliable predictor for the outcome of patients admitted to the intensive care unit (ICU) [41]. In line with the literature, septic patients within the presented investigation revealed significantly decreased platelet counts in comparison with healthy volunteers, whereas septic patients suffering from overt DIC revealed the lowest platelet counts [5, 42–45]. Besides decreased platelet counts, we were able to provide evidence for impaired platelet function in sepsis as compared to both control groups. Sepsis-associated changes in arachidonic acid metabolism might serve as an explanation for the observed impairments of platelet function in septic patients. Therefore, further examinations via an LC–MS-based approach for the determination of 11d-TX B_2_ (a stable and inactive metabolite of TXA2) and PG F1α (a stable and inactive metabolite of PGI2) were performed. TXA2 and PGI2 are supposed to be of importance in vascular haemostasis as well as homoeostasis due to their opposing effects on vasoactivity and platelet aggregation [46–48]. On the one hand, PGI2 is known to be a potent inhibitor of platelet aggregation and acts as a vasodilator. On the other, TXA2 induces platelet aggregation and acts as a potent vasoconstrictor. Since these two eicosanoids (both representing COX-dependent metabolites of arachidonic acid) are quite unstable, their inactive and stable hydrolysis products 11d-TX B_2_ and PG F1α need to be determined as indirect surrogates for the TXA2 and PGI2 loads in different inflammatory settings [47, 49–51]. In summary, the present investigation revealed a strong upregulation of the anti-aggregatory PG F1α (as a surrogate for PGI2), whereas the proaggregatory 11d-TX B_2_ (as a surrogate for TXA2) was shown to be downregulated at sepsis onset, resulting in strongly impaired haemostatic platelet function. Therefore, Yaguchi et al. already hypothesized that sepsis seems to induce a redirection of platelet function from haemostasis towards other non-haemostatic functions [45, 52].

Septic coagulopathy is a complex and dynamic disease entity, tilting from an initial hypercoagulopathy in non-overt DIC patients towards a hypocoagulable disease state in overt DIC patients, especially at later stages. However, the specific temporal kinetics of septic coagulopathy is hallmarked by a high interindividual variability and an assessment of initial hypercoagulability by the use of standard coagulation measurements is not feasible. What all this amounts to, is that up to now one cannot reliably predict the critical turning point from hypercoagulability to hypocoagulability in the individual patient in daily clinical routine. This problem might be solved by the use of modern viscoelastic POCT devices, since an initial hypercoagulability due to lysis shutdown, as indicated by an increased LI in viscoelastic POCT, in the presence of other viscoelastic signs for hypercoagulability (e.g. shortened CFT, etc.) might be used for the identification of hypercoagulable septic patients likely to benefit from an anticoagulant treatment.

To the best of our knowledge, this is the first clinical study assessing detailed pathomechanisms, the specific temporal kinetics and the outcome relevance of septic coagulopathy at very early stages after sepsis onset by the use of a sophisticated coagulation monitoring (including routine coagulation parameters, viscoelastic and aggregometric POCT, TGA, as well as an LC–MS- and ELISA-based evaluation of septic coagulopathy).

## Limitations

The following limitations need to be addressed in connection with the presented manuscript. The study was conducted in terms of a monocentric project, including only a limited number of patients. In the surgical control group, only patients undergoing major abdominal surgery were enrolled. Moreover, those patients most frequently suffered from a tumorous disease. This might have influenced the results, since tumour cells are able to activate blood coagulation through multiple mechanisms [53]. Accordingly, D-dimers were shown to be increased in surgical patients prior to the surgical procedure in comparison with healthy controls. The viscoelastic tests used within the presented investigation might have been not sensitive enough to identify minor changes within the fibrinolytic homoeostasis, so that a tPA-activated thromboelastometrical assay would have been of value for the assessment of the magnitude of an ongoing fibrinolysis shutdown. Besides, this decreased ability to detect minor changes is not only restricted to the evaluation of the fibrinolytic system, since the great amount of activator used in the different thromboelastometrical assays shifts the balance towards an over-activation in general. Furthermore, most patients in the septic group revealed an underlying abdominal focus, so that it has to be questioned critically whether these results can be transferred to septic patients with other foci without any limitations. Moreover, it cannot be fully excluded that the low-dose UFH treatment regime in septic as well as surgical patients might have had an influence on the presented test results, even though UFH and enoxaparin calibrated anti-Xa activity as well as thrombin time showed no significant influence. Besides, since the study was designed for a maximal observation period of 30 days, conclusions regarding long-term outcome cannot be drawn.

## Conclusions

Septic patients were shown to be hallmarked by an excessive activation of the coagulation system, resulting in a consumption of pro- and anticoagulatory factors. An acute fibrinolysis shutdown occurred early after sepsis onset, as assessed by viscoelastic POCT, which was associated with an increased morbidity as well as mortality. Due to the consumption of coagulation factors and platelets, this initial hypercoagulability in non-overt DIC patients is tilting towards an overt DIC with a high risk of bleeding disorders at later stages. Our current findings clearly support the use of viscoelastic POCT in sepsis in order to stratify affected patients into hypo- and hypercoagulable, which might probably be used for the identification of hypercoagulable non-overt DIC patients likely to benefit from an anticoagulant treatment.

## Additional files

**Additional file 1.** Subgroup analyses with septic patients suffering from DIC or not (non-DIC) at sepsis onset. Data are presented by median and interquartile range (Q1–Q3). A *P* value < 0.05 was considered statistically significant. Concerning symbolism and higher orders of significance: p < 0.05: *, p < 0.01: **, p < 0.001: ***. Abbreviations: CT, clotting time; LI, lysis index.

**Additional file 2.** Subgroup analyses with deceased and surviving septic patients 30 days after sepsis onset. Data are presented by median and interquartile range (Q1–Q3). A *P* value < 0.05 was considered statistically significant. Concerning symbolism and higher orders of significance: p < 0.05: *, p < 0.01: **, p < 0.001: ***. Abbreviations: CT, clotting time; CFT, clot formation time; LI, lysis index; AUC, area under the curve.

## Abbreviations

PG F1α: 6-keto-prostaglandin F1α
11d-TX B_2_: 11-dehydro-thromboxane B_2_
aPTT: activated partial thromboplastin time
ADP: adenosine diphosphate
AT: antithrombin
AUC: area under the curve
CLP: cecal ligation and puncture
CT: clotting time
CFT: clot formation time
CE: collision voltage
CV: cone voltage
COX: cyclooxygenase
CRP: C-reactive protein
DIC: disseminated intravascular coagulation
ETP: endogenous thrombin potential
ELISA: enzyme-linked immunosorbent assay
tPA: free tissue plasminogen activator
HPLC: high-performance liquid chromatography
INR: international normalized ratio
ISTH: International Society on Thrombosis and Haemostasis
LMWH: low molecular weight heparin
LPS: lipopolysaccharide
LC–MS: liquid chromatography–mass spectrometry
LLE: liquid–liquid extraction
MCF: maximal clot firmness
MEA: multiple electrode aggregometry
MRM: multiple reaction monitoring
NATEM: non-activated TEM
NSAIDs: non-steroidal anti-inflammatory drugs
PAMP: pathogen-associated molecular pattern
PRR: pattern recognition receptor
PAI-1: plasminogen activator inhibitor 1
PPP: platelet-poor plasma
POCT: point-of-care testing
PCT: procalcitonin
PGI2: prostacyclin
PT: prothrombin
ROC: receiver operating characteristic
*R*_*t*_: retention time
SOFA: sepsis-related organ failure assessment
TGA: thrombin generation assay
TAT: thrombin–antithrombin complex
TRAP: thrombin receptor-activating peptide
LI: thromboelastometrical lysis indices
TXA2: thromboxane A2
TF: tissue factor
total tPA: total tissue plasminogen activator
UFH: unfractionated heparin
VTE: venous thromboembolism
