# Supplementary material for: Acute fibrinolysis shutdown occurs early in septic shock and is associated with increased morbidity and mortality: results of an observational pilot study
Source: Ann Intensive Care. 2019 Jan 30;9:19. doi: 10.1186/s13613-019-0499-6 (PMC6353981; doi:10.1186/s13613-019-0499-6)
Supplement: Supplementary file 1 — Additional file 1. Subgroup analyses with septic patients suffering from DIC or not (non-DIC) at sepsis onset. Data are presented by median and interquartile range (Q1–Q3). A P value < 0.05 was considered statistically significant. Concerning symbolism and higher orders of significance: p < 0.05: *, p < 0.01: **, p < 0.001: ***. Abbreviations: CT, clotting time; LI, lysis index. [file 13613_2019_499_MOESM1_ESM.docx]

**Additional file 1.** Subgroup analyses

| **Timepoint** | **onset** | | |
| --- | --- | --- | --- |
| **EXTEM-Test** | | | |
|  | **healthy** | **Non-DIC** | **DIC** |
| **CT [sec]** | 57.0 (54.0-60.0) | 73.0 (67.0-86.0) | 88.0 (82.0-93.0) |
| **(normal range: 38-79)** | **<0.001***** | |  |
|  | **<0.001***** | | |
|  |  | **0.022*** | |
| **CFT [sec]** | 77.5 (63.3-83.8) | 54.0 (51.0-72.0) | 72.0 (47.0-103.0) |
| **(normal range: 34-159)** | **0.002**** | |  |
|  | 0.500 | | |
|  |  | **0.006**** | |
| **LI 45 min [%]** | 96.0 (94.0-98.0) | 99.0 (98.0-100) | 100 (99.0-100) |
| **(normal range: >85)** | **<0.001***** | |  |
|  | **<0.001***** | | |
|  |  | 0.082 | |
| **LI 60 min [%]** | 92.0 (90.0-93.75) | 97.0 (95.5-97.5) | 98.5 (97.3-99.0) |
| **(normal range: >85)** | **<0.001***** | |  |
|  | **<0.001***** | | |
|  |  | 0.141 | |
| Data are presented by median and interquartile range (Q1–Q3).  A p-value < 0.05 was considered statistically significant. Concerning symbolism and higher orders of significance: p < 0.05: *, p < 0.01: **, p < 0.001: ***. Abbreviations: CT, clotting time; CFT, clot formation time; LI, lysis index | | | |
