# Supplementary material for: Acute fibrinolysis shutdown occurs early in septic shock and is associated with increased morbidity and mortality: results of an observational pilot study
Source: Ann Intensive Care. 2019 Jan 30;9:19. doi: 10.1186/s13613-019-0499-6 (PMC6353981; doi:10.1186/s13613-019-0499-6)
Supplement: Supplementary file 2 — Additional file 2. Subgroup analyses with deceased and surviving septic patients 30 days after sepsis onset. Data are presented by median and interquartile range (Q1–Q3). A P value < 0.05 was considered statistically significant. Concerning symbolism and higher orders of significance: p < 0.05: *, p < 0.01: **, p < 0.001: ***. Abbreviations: CT, clotting time; CFT, clot formation time; LI, lysis index; AUC, area under the curve. [file 13613_2019_499_MOESM2_ESM.docx]

**Additional file 2.** Subgroup analyses

| **Timepoints** | **Onset** | | **3h** | | **6h** | | **12h** | | **24h** | | **48h** | | **7d** | |
| --- | --- | --- | --- | --- | --- | --- | --- | --- | --- | --- | --- | --- | --- | --- |
| **EXTEM-Test** | | | | | | | | | | | | | | |
| **Groups** | **Deceased** | **Survivor** | **Deceased** | **Survivor** | **Deceased** | **Survivor** | **Deceased** | **Survivor** | **Deceased** | **Survivor** | **Deceased** | **Survivor** | **Deceased** | **Survivor** |
| **LI 60 min [%]**  **(normal range: >85%)** | 98.5 (97.3-99.0) | 97.0 (96.0-97.5) | 99.0 (96.3-100.0) | 96.5 (95.3-98.8) | 99.0 (98.0-99.8) | 96.0 (96.0-98.0) | 99.5 (96.5-100.0) | 96.0 (95.0-98.0) | 99.0 (97.0-100.0) | 96.0 (94.5-98.5) | 100.0 (98.8-100.0) | 96.0 (95.0-99.0) | 98.5 (98.0-99.3) | 97.0 (93.8-97.8) |
|  | 0.130 | | 0.217 | | **0.018*** | | **0.043*** | | **0.033*** | | **0.027*** | | **0.045*** | |
| Data are presented by median and interquartile range (Q1–Q3). A p-value < 0.05 was considered statistically significant. Concerning symbolism and higher orders of significance: p < 0.05: *, p < 0.01: **, p < 0.001: ***, Abbreviations: CT, clotting time; CFT, clot formation time; LI, lysis index; | | | | | | | | | | | | | | |
|  |  |  |  |  |  |  |  |  |  |  |  |  |  |  |
| **Timepoints** | **Onset** | | **3h** | | **6h** | | **12h** | | **24h** | | **48h** | | **7d** | |
| **Groups** | **Deceased** | **Survivor** | **Deceased** | **Survivor** | **Deceased** | **Survivor** | **Deceased** | **Survivor** | **Deceased** | **Survivor** | **Deceased** | **Survivor** | **Deceased** | **Survivor** |
| **ADP-Test** | | | | | | | | | | | | | | |
| **AUC [AU*min]**    **(normal range: 53-122)** | 24.5 (16.0-33.3) | 38.5 (24.3-68.3) | 23.0 (19.0-36.0) | 45.0 (21.0-64.0) | 16.5 (11.8-43.3) | 44.0 (32.3-66.3) | 21.0 (10.0-57.5) | 63.0 (33.8-74.5) | 17.0 (11.5-40.5) | 46.0 (28.5-74.3) | 13.0 (11.0-21.0) | 36.0 (20.0-66.0) | 47.5 (26.3-68.0) | 86.0 (42.5-130.5) |
|  | 0.108 | | 0.183 | | **0.025*** | | 0.076 | | **0.014*** | | **0.049*** | | 0.170 | |
| **ASPI-Test** | | | | | | | | | | | | | | |
| **AUC [AU*min]**    **(normal range: 75-136)** | 24.5 (15.8-75.0) | 77.5 (46.0-92.0) | 26.0 (15.5-53.5) | 77.0 (40.0-106.0) | 33.0 (12.0-41.3) | 88.5 (49.8-128.0) | 29.0 (12.0-47.0) | 88.5 (45.3-114.5) | 26.0 (18.0-61.5) | 82.0 (50.5-131.8) | 23.0 (17.0-51.0) | 85.0 (46.0-122.0) | 91.5 (42.3-152.0) | 102.0 (84.5-141.5) |
|  | 0.083 | | 0.071 | | **0.002**** | | **0.006**** | | **0.009**** | | **0.022*** | | 0.896 | |
| **COL-Test** | | | | | | | | | | | | | | |
| **AUC [AU*min]**    **(normal range: 46-117)** | 34.0 (22.5-43.0) | 59.0 (39.0-76.8) | 29.0 (23.0-45.8) | 80.0 (53.0-117.0) | 23.5 (16.3-73.3) | 80.0 (41.3-106.8) | 29.0  (8.5-68) | 81.5 (55.5-116.8) | 38.0 (21.5-79.0) | 76.0 (44.0-96.8) | 40.0 (23.0-52.0) | 65.0 (38.0-106.0) | 42.5 (35.8-63.8) | 70.0 (52.5-120.5) |
|  | **0.022*** | | 0.060 | | **0.018*** | | **0.017*** | | 0.050 | | **0.046*** | | 0.214 | |
| **TRAP-Test** | | | | | | | | | | | | | | |
| **AUC [AU*min]**    **(normal range: 94-156)** | 44.5 (30.8-56.8) | 79.5 (57.8-108.3) | 40.5 (36.0-53.0) | 57.0 (49.0-97.0) | 35.0 (26.5-66.0) | 85.0 (69.3-106.3) | 34.0 (18.0-89.0) | 79.0 (51.8-104.3) | 51.0 (27.0-75.0) | 78.5 (46.5-98.3) | 31.0 (18.5-42.3) | 62.0 (45.0-123.0) | 89.0 (74.5-109.0) | 142.0 (112.0-172.0) |
|  | **0.005**** | | 0.071 | | **0.002**** | | 0.088 | | 0.096 | | **0.014*** | | 0.090 | |
| Data are presented by median and interquartile range (Q1–Q3). A p-value < 0.05 was considered statistically significant. Concerning symbolism and higher orders of significance: p < 0.05: *, p < 0.01: **, p < 0.001: ***,  Abbreviations: AUC, area under the curve | | | | | | | | | | | | | | |
